# Simulation of the Strain Amplification in Sulci Due to Blunt Impact to the Head

**DOI:** 10.3389/fneur.2020.00998

**Published:** 2020-09-08

**Authors:** Brian T. Fagan, Sikhanda S. Satapathy, J. Neal Rutledge, Steven E. Kornguth

**Affiliations:** ^1^U.S. Army Combat Capabilities Development Command - Army Research Laboratory, Aberdeen Proving Ground, MD, United States; ^2^Austin Radiological Association, Austin, TX, United States; ^3^Dell Medical School, University of Texas at Austin, Austin, TX, United States; ^4^Department of Kinesiology and Health Education, University of Texas at Austin, Austin, TX, United States

**Keywords:** injury biomechanics, finite element analysis, computational biomechanics, traumatic brain injury, blunt impact to head

## Abstract

Traumatic brain injury (TBI) has become a concern in sports, automobile accidents and combat operations. A better understanding of the mechanics leading to a TBI is required to cope with both the short-term life-threatening effects and long-term effects of TBIs, such as the development chronic traumatic encephalopathy (CTE). Kornguth et al. (1) proposed that an inflammatory and autoimmune process initiated by a water hammer effect at the bases of the sulci of the brain is a mechanism of TBI leading to CTE. A major objective of this study is to investigate whether the water hammer effect is present due to blunt impacts through the use of computational models. Frontal blunt impacts were simulated with 2D finite element models developed to capture the biofidelic geometry of a human head. The models utilized the Arbitrary Lagrangian Eulerian (ALE) method to model a layer of cerebrospinal fluid (CSF) as a deforming fluid allowing for CSF to move in and out of sulci. During the simulated impacts, CSF was not observed to be driven into the sulci during the transient response. However, elevated shear strain levels near the base of the sulci were exhibited. Further, increased shear strain was present when differentiation between white and gray matter was taken into account. Both of the results support clinical observations of (1).

## Introduction

The marked increase in incidence of Traumatic Brain Injury (TBI) in athletes engaged in contact sports and Soldiers returning from deployment over the past two decades has led to extensive research regarding the mechanisms causing this injury and potential treatments for the condition (1–8). In the affected population, inflammatory changes in the brain including activation of microglia, generation of antibodies to neuronal and glial proteins, increased permeability of the blood brain barrier and changes in the expression of interleukins and cytokines are associated with the clinical status of the subject (1, 9, 10). These changes frequently occur ten or more years after injuries have occurred. Neuropathological studies on brain samples recovered from patients who died following diagnosis with severe TBI were found to have changes in the brain parenchyma that are designated as Chronic Traumatic Encephalopathy (CTE) (7, 11). CTE is currently a diagnosis that is obtained post mortem and is dependent upon brain tissue samples that are examined microscopically. McKee and colleagues define CTE as a “tauopathy, characterized by the deposition of hyperphosphorylated tau (p-tau) protein as neurofibrillary tangles, astrocytic tangles, and neurites in striking clusters around small blood vessels of the cortex, typically at the sulcal depths” (11). Of primary interest to the current study is the observation that the lesions in CTE cluster around small vessels of the cortex localized at the sulcal depths rather than at the apices of the gyri. During rapid deceleration of the head associated with forceful impacts, the apices of the gyri are the sites of initial contact with the calvarium and these regions might be anticipated to be the major site of injury; this is not the case, however. In an earlier report, Kornguth et al. (1) proposed that the depths of the sulci will be the primary site of injury from traumatic head impacts as a result of a “water hammer” effect following the forceful impact of the brain upon a rigid bone (calvarium) and the incompressibility of the cerebral spinal fluid (water) that is driven into the sulci where the energy is dissipated at the base of the sulcus. The resulting forces at the base of the sulci were hypothesized to cause shearing at the base, thereby causing tears in the regional microvessels with microbleeds as a consequence. We undertook a computational modeling study to investigate the load transmission to the brain under blunt impact conditions and understand the load amplification mechanism at the base of the sulcus as observed in Kornguth et al. (1).

Various computational models of the human head exist in the literature with various degrees of sophistication to analyze load transfer under blunt, blast and ballistic conditions [Ho and Kleiven et al. (12), Panzer et al. (13), Kraft et al. (14), and Zhang et al. (15)]. Cloots et al. (16) exercised a representative model of the morphological heterogeneity of the cerebral cortex, and found that the heterogeneities had an influence on the equivalent stress distribution. The maximum equivalent stress in Cloots' heterogeneous model was found to increase by a factor of about 1.3–1.9 compared to the homogeneous model. Ho and Kleiven (12) included sulci in a brain model to simulate inertial loading effects using the Lagrangian finite element method. Their study demonstrated that the inclusion of sulci alters both the strain and strain distribution and argued that it was connected to injury observed in cases with diffuse axonal injury (DAI). Typically Lagrangian methods, where various head regions are represented by finite elements of fixed connectivity, are used. While such methods work well for loading conditions causing small and some large deformations in the head components, they are not well-suited to the large mesh distortion associated with the flow of fluid like materials. Alternate computational schemes such as Eulerian and Arbitrary Lagrangian Eulerian (ALE) methods are more suitable to study the large deformation expected in cerebrospinal fluid (CSF) material in the current study. We employed the ALE method, where skull and brain components are modeled using Lagrangian finite elements, whereas the CSF is modeled using an Eulerian description.

The goal of this study is to examine the effects of brain geometry on impact-induced stress propagation, especially at the base of the sulci, and examine the origin of structural changes in this region as reported by Kornguth et al. (1).

## Numerical Model

**Figure 1:**
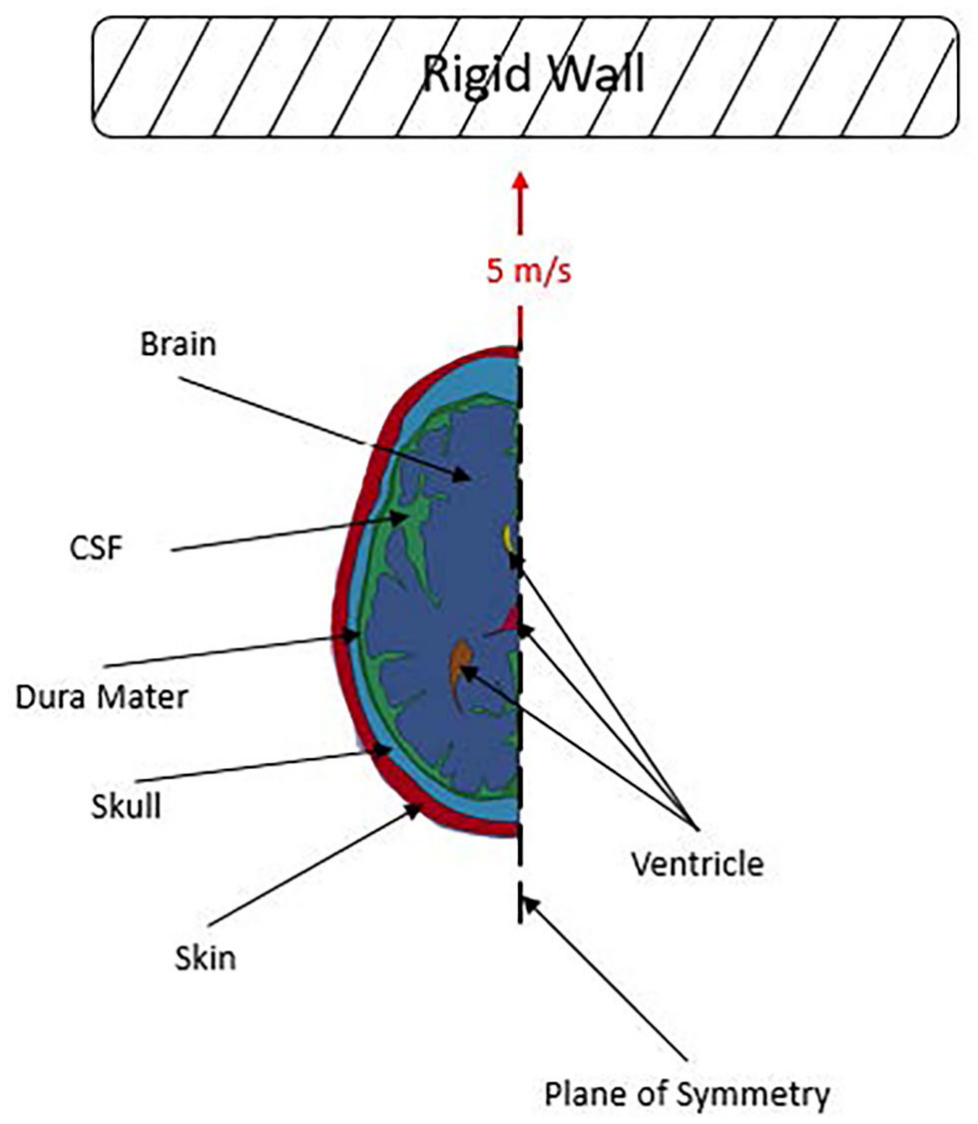
Simulation setup.

To investigate Kornguth et al.'s (1) proposal, 2D finite element models of a human head were developed. 2D finite element models of heads are commonly used in literature to study various aspects of TBI and the responses of heads to various loading conditions (13, 16, 17). The models used in this study were designed to simulate a human head impacting a rigid wall at a prescribed initial velocity with various brain-structure approximations: a smooth brain and a set of brains with sulci and gyri with and without white and gray matter differentiation. The displacement, stress and strain in the head arising from the impact event were computed. A schematic of the simulation setup is shown in Figure 1, which illustrates a 5 m/s linear frontal impact into a rigid wall.

The finite element code LS-DYNA was used to model the impact event. The ALE module of LS-DYNA was selected as it provides a means to treat the CSF as a deforming fluid while treating the skull and the brain as deforming solids. This method is better suited to model the fluid-structure interaction (FSI) between the CSF and the adjacent skull and brain in comparison to a purely Lagrangian method. The FSI between the fluid (CSF) and the structure (head) was modeled using the LS-DYNA keyword ^*^CONSTRAINED_LAGRANGIAN_IN_SOLID. This keyword utilizes a penalty-based method for coupling, and in this study the option DIREC=1 was used, which couples under both compression and tension. Further, this option does not inhibit tangential motion of the material on either side of the interface.

### Model Geometry

The head geometry used in this study was modified from Zygote Media Group, Inc.'s, 2015 human male solid model generation II (Zygote Media Group Inc., American Fork, UT). In this model the Skull was developed from CT scans of a single individual. The nervous system model was developed from 3D MR scans of a second individual. The models were merged together by Zygote following their in house procedures (18). The scan were taken from “individuals selected for conformation to anthropomorphic parameters of a 50th percentile U.S. individual rather than for visual aesthetics alone” (18). A transverse slice was taken to generate the desired 2D geometry. The plane chosen included the cingulate region of the brain as it has been shown that the anterior region of the cingulate is the most vulnerable to mild TBI (19). This layer was chosen based on features of clinical interest associated with the water-hammer hypothesis in Kornguth et al.'s paper (1). The depths of the sulci in plane are similar to those depths reported previously for the brain (20, 21). The widths of the sulci in the model we used appear similar to that reported by Kochunov [**Figure 5** of (20)]. Additionally, the chosen plane has sulci that are oriented favorably to exhibit the water hammer hypothesis from a frontal impact. Further, only one half of the geometry was modeled to take advantage of the mid-sagittal symmetry of the problem.

**Figure 2:**
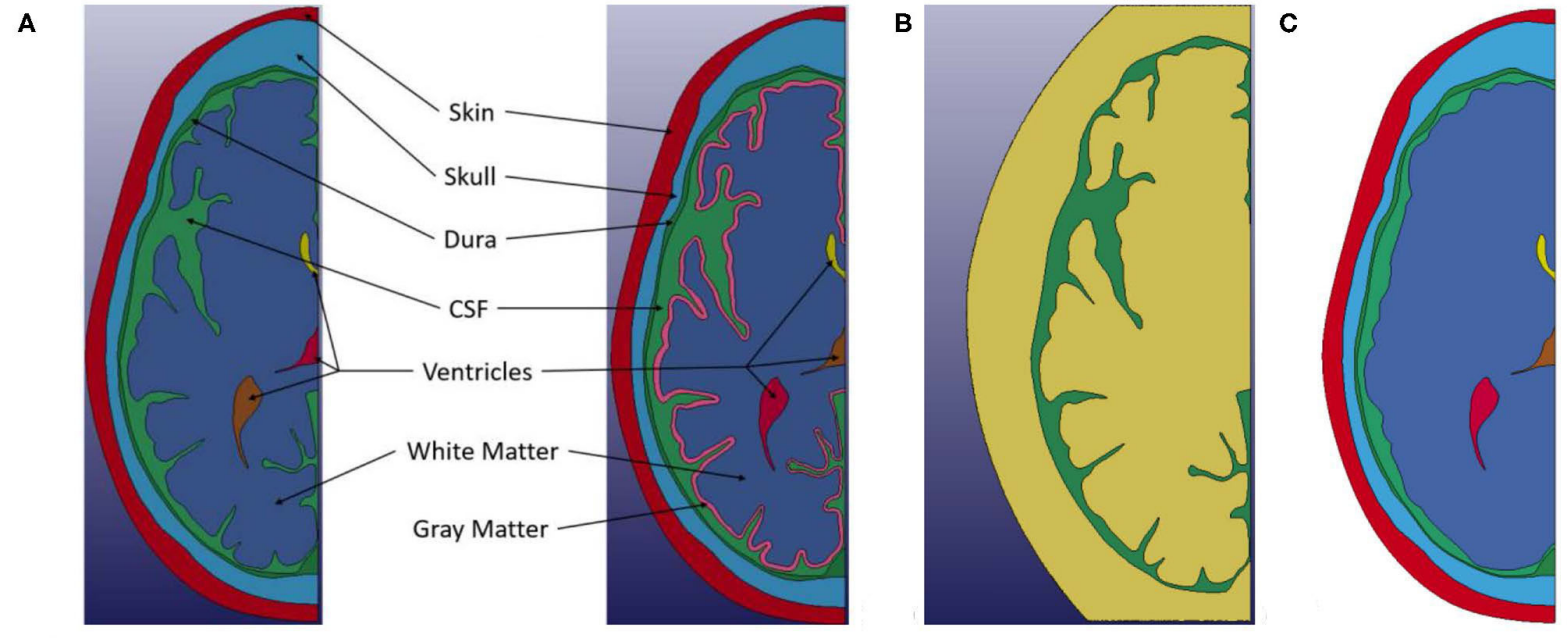
**(A)** Left Geometry of non-differentiated model. **(A)** Right Geometry of the white and gray matter differentiated model. **(B)** ALE computational domain (Green) Initial region occupied by CSF material. (Yellow) Initial empty region. Only the CSF material interacts with the Lagrangian parts, but it is able to move and deform within the combined area of the Green and Yellow meshes. **(C)** Smooth brain model.

Two primary finite element models are presented in this study: one with differentiation between white and gray matter and the other without such differentiation, where ALE formulation was used for CSF and Lagrangian formulation was used for all other components. Two additional models, one using purely Lagrangian formulation, and another using a smooth brain with ALE formulation for CSF were also used to compare the effects of numerical and geometry approximations. Within each model, there are five common components: skin, bone, dura mater, CSF and ventricles as shown in Figure 2A. The brain geometry is also the same for each model, except for the smooth brain model where the sulci were filled with brain material. In the differentiated model, an ~3-mm layer is inset from exterior surface of the brain geometry to represent the covering of cerebral cortex by gray matter, which is approximately the average thickness reported for gray matter (22). This distinction in the models is shown in Figure 2A. The ALE domain for the model is shown in Figure 2B with green representing the initial region of the CSF in the domain and yellow illustrating the initially empty region of the fluid domain. Only the CSF material interacts with the Lagrangian parts, but it is able to move and deform within the combined area of the Green and Yellow regions. The falx cerebri was not modeled in this study. This membrane is known to play a supportive role for the brain and can impart rotational forces onto the brain. Thus, it has been included in previous finite element models of the head (23). Deformation in the brain can be caused from both translational and rotational motions. However, a linear frontal impact was used in this study to examine the proposed water-hammer effect, so the primary motion in this study would be translational. Thus, the assumption was made that the presence of this membrane was not expected to significantly influence the deformation resulting from a linear frontal impact on a 2D transverse section of the head. As such, it was not included in the model.

### Model Setup

The models were composed of a single layer of hexahedral elements in the out-of-plane (thickness, z-direction) direction with plane strain conditions applied. To achieve a plane strain condition for the model the nodes on each side of the single layer was constrained so that displacement in the z-direction is zero. The head (skin/bone/dura/brain/ventricles) was discretized into about 200,000 elements for both models. Nodes were shared at the interfaces of the skin, bone, and dura mater, and also in between the ventricles and brain. Consequently, the nodal displacement of the materials at the interfaces are the same, yet the adjacent elements are free to deform independently, allowing for separate strain (and stress) to develop across the interface. On the other hand, the CSF can deform and separate from adjacent materials since it is treated as an ALE part.

The ALE background mesh contains about 240,000 hexahedral elements in the models. The ^*^MAT_VACUUM and ^*^ALE_MULTI-MATERIAL_GROUP keywords were used to assign properties to the initial empty region of the ALE domain. This in essence treats the region as if no material is present.

In both models, a self-contact algorithm was applied to the brain. The algorithm checks for contact between outer surfaces of the brain with its other outer surfaces. This was done to accurately capture the self-contact effects and prevent self-penetration on each side of the sulci as the CSF is forced from the sulci bases during deformation.

Further, symmetry boundary conditions were applied along the mid-sagittal plane, and an initial velocity of 5 m/s was applied to all of the parts. A planar rigid wall in LS-DYNA was then placed close to the front of each head model and aligned for a frontal impact. These conditions were chosen to represent a potential head loading condition for impact between two soccer players or an impact between a player and the ground. Soccer players at both a competitive and non-competitive level can reach speeds of >5 m/s while sprinting (24). A representative soccer related impact rate was chosen for this study since Kornguth et al.'s (1) findings were based on a clinical study of collegiate soccer players. In addition, the linear frontal impact was chosen as it was assumed that based on the orientation and location of the sulci in our model a frontal impact would be a good candidate to investigate Kornguth et al.'s hypothesized water hammer effect.

In addition to the two primary models presented in this study, two additional models were developed to investigate the effects of a Lagrangian treatment of CSF and the use of an ALE CSF without a convoluted brain. Both of these additional models were developed from the non-differentiated brain geometry. The first model treated the CSF in a Lagrangian fashion. In this model both the interfaces between the dura mater and the CSF, and the CSF and brain had shared nodes between the parts. The second additional model was developed to investigate the combination of a smoothed-brain (i.e., convolutions removed) and ALE CSF. To generate this model, the sulci were filled using a spline between the tops of the adjacent gyri, and the brain material property was assigned to the new region, see Figure 2C.

### Constitutive Models

In this study, the skin was modeled as a linear elastic material. The density, Young's modulus, and Poisson's ratio used in our model were ρ = 1,130 kg/m^3^, *E* = 16.7 MPa, and ν = 0.42, respectively (25).

An elastic constitutive model was also used for the cranial bone. A density of 1,710 kg/m^3^, Young's modulus of 3.4 GPa and Poisson's ratio of 0.19 were used in our model (26, 27). Material failure of the bone was not modeled in this study.

Similarly, the dura mater was modeled with an elastic model with the parameters ρ = 1,133 kg/m^3^, *E* = 31.5 MPa, and ν = 0.45 (28).

In literature, the brain has frequently been reported to exhibit viscoelastic mechanical responses (29, 30) and hence was modeled in this study with a viscoelastic constitutive model (31). For the white matter, the properties in the model were ρ = 1,040 kg/m^3^, *K* = 2.19 GPa, G_0_ = 41 KPa, G_∞_ = 7.8 KPa and β = 400 s^−1^ where K, G_0_, G_∞_, and β are bulk modulus, short-term shear modulus, long-term shear modulus, and decay constant, respectively. The properties for gray matter were ρ = 1,040 kg/m^3^, *K* = 2.19 GPa, G_0_ = 34 KPa, G_∞_ = 6.4 KPa, and β = 400s^−1^. For the non-differentiated model, the parameters for white matter were used for the entire brain.

The ventricles were modeled as an elastic fluid using LS-DYNA's ^*^MAT_ELASTIC_FLUID in which the shear modulus was set to zero. Parameters for water were used: density, ρ = 1,000 kg/m^3^ and bulk modulus, *K* = 2.15 GPa. Additionally, a viscosity coefficient of 0.1 was used.

The Gruneisen equation of state (EOS) was used to model the volumetric response of the CSF. Specifically, the properties for water were used for the CSF. These are ρ = 1,000 kg/m^3^, *C* = 1,484 m/s, *S*_1_ = 19.79 and γ_0_ = 0.11, where ρ is density, C is the bulk sound speed, S_1_ is the coefficient for the Hugoniot slope of particle-velocity and shock-velocity curve, and γ_0_ is the Gruneisen constant, respectively (13).

For this study, a CSF cavitation pressure of −100 kPa was examined. The exact cavitation pressure of water can vary based on both its state (i.e., temperature; presence of gas and level of gas saturation) and the presence of nucleation sites. Pressure values for the cavitation of water reported in literature have a large variation even between similar experimental setups (32). Typical values experimentally reported for acoustic cavitation fall within −1 MPa to 0.1 MPa (33). A cavitation threshold pressure for CSF of −100 kPa has been used in several analyses of finite element head models (13, 34). The CSF cavitation pressure in essence represents the tensile limit of the material. The cavitation pressure value only acts as the negative pressure limit that the CSF can experience. A summary of all the constitutive models used in this study can be found in Table 1.

**Table 1:** Material properties used in the head model.

**Component**	**Model**	**Properties**
Skin (25)	Elastic	ρ = 1,130 kg/m^3^ *E* = 16.7 MPa υ = 0.499
Skull (26, 27)	Elastic	ρ = 1,710 kg/m^3^ *E* = 3.4 GPa υ = 0.19
Dura mater (28)	Elastic	ρ = 1,133 kg/ m^3^ *E* = 32.5 MPa υ = 0.45
White matter (31)	Viscoelastic	ρ = 1,040 kg/m^3^ *K* = 2.19 GPa *G*_0_= 41 kPa *G*_∞_= 7.8 kPa β = 700/s
Gray matter (31)	Viscoelastic	ρ = 1,040 kg/m^3^ *K* = 2.19 GPa *G*_0_ = 34 kPa *G*_∞_= 6.4 kPa β = 400/s
Ventricles	Elastic fluid	ρ = 1,000 kg/m^3^ *K* = 2.15 GPa Viscosity coef. = 0.1
CSF (13)	Gruneisen EOS	ρ = 1,000 kg/m^3^ *C* = 1,484 m/s *S*_1_ = 19.79 γ_0_ = 0.11 Cavitation pressure = −0.1 MPa

## Results

### Non-differentiated Brain Model

**Figure 3:**
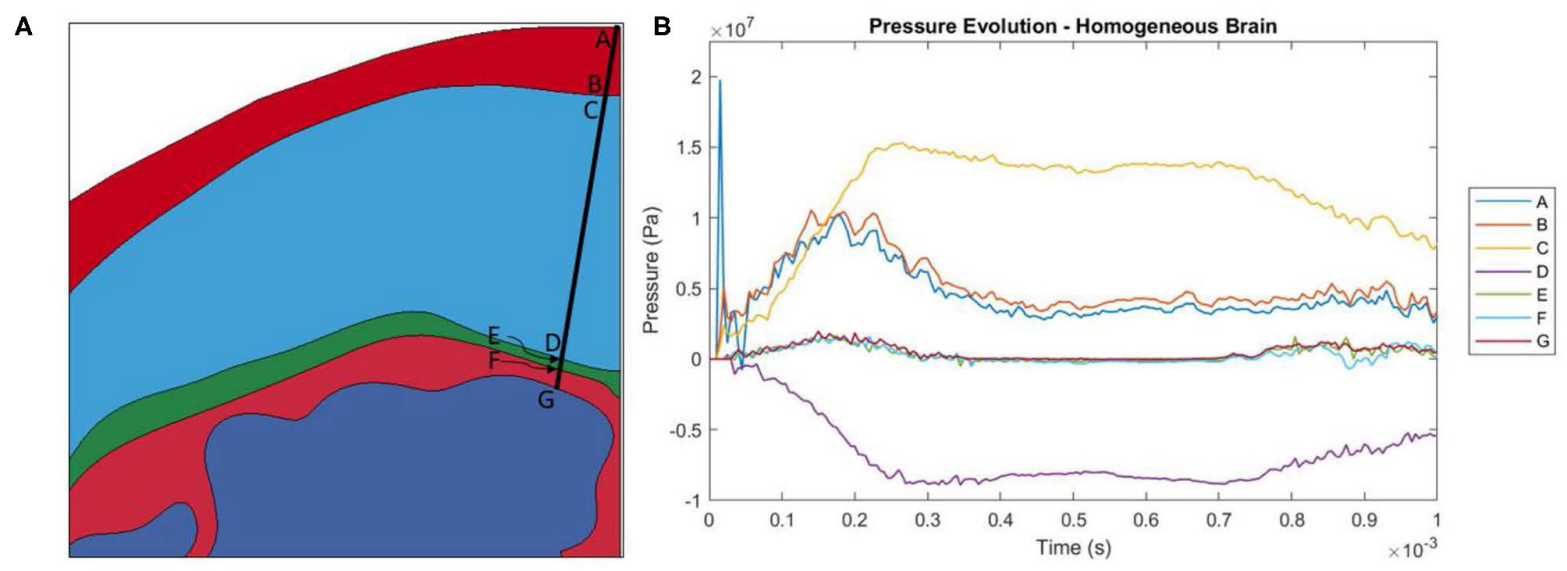
**(A)** Locations at which pressure history was measured. Locations A and B are the locations where the pressure wave enters and leaves the skin. C and D are the locations where the pressure wave enters and leaves the skull. E and F are the locations where the pressure wave enters and leaves the dura mater. Finally, G is where the pressure wave enters the brain. **(B)** Evolution of pressure in the head model with a homogeneous brain.

As the head impacts the rigid wall, a pressure wave is generated that propagates away from the contact region through skull and CSF into brain. This compression wave causes progressive deceleration of the head components. As the pressure wave transmits through the material interfaces, depending on the angle of incidence of the wave-front with the interface, the pressure wave partially transmits the compression, generates shear stress and partly reflects as tension. The time-history of the pressure on each side of each interface is shown in Figure 3B. Since the symmetry plane contains the median longitudinal fissure, we chose to plot the pressure along a line slightly at an angle (10 degrees) to the symmetry plane, as shown in Figure 3A. The stress wave reverberation in the skin is exhibited by pressure on the skull side lagging the pressure at the impact point. The skin side of the skull shows large compressive stress whereas the brain side of the skull shows large tensile stress, indicative of skull flexure. The stress amplitude attenuates significantly before it reaches the brain.

**Figure 4:**
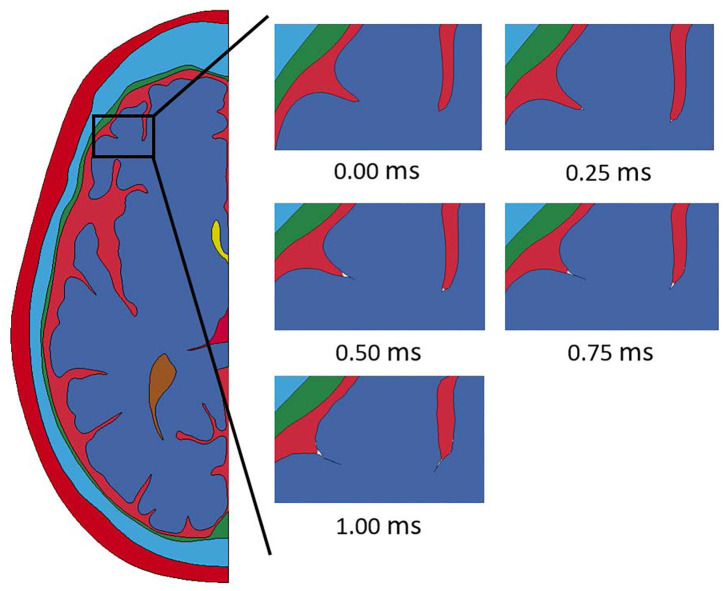
Closure of sulci throughout the duration of the impact event modeled with a homogeneous brain homogeneous brain.

**Figure 5:**
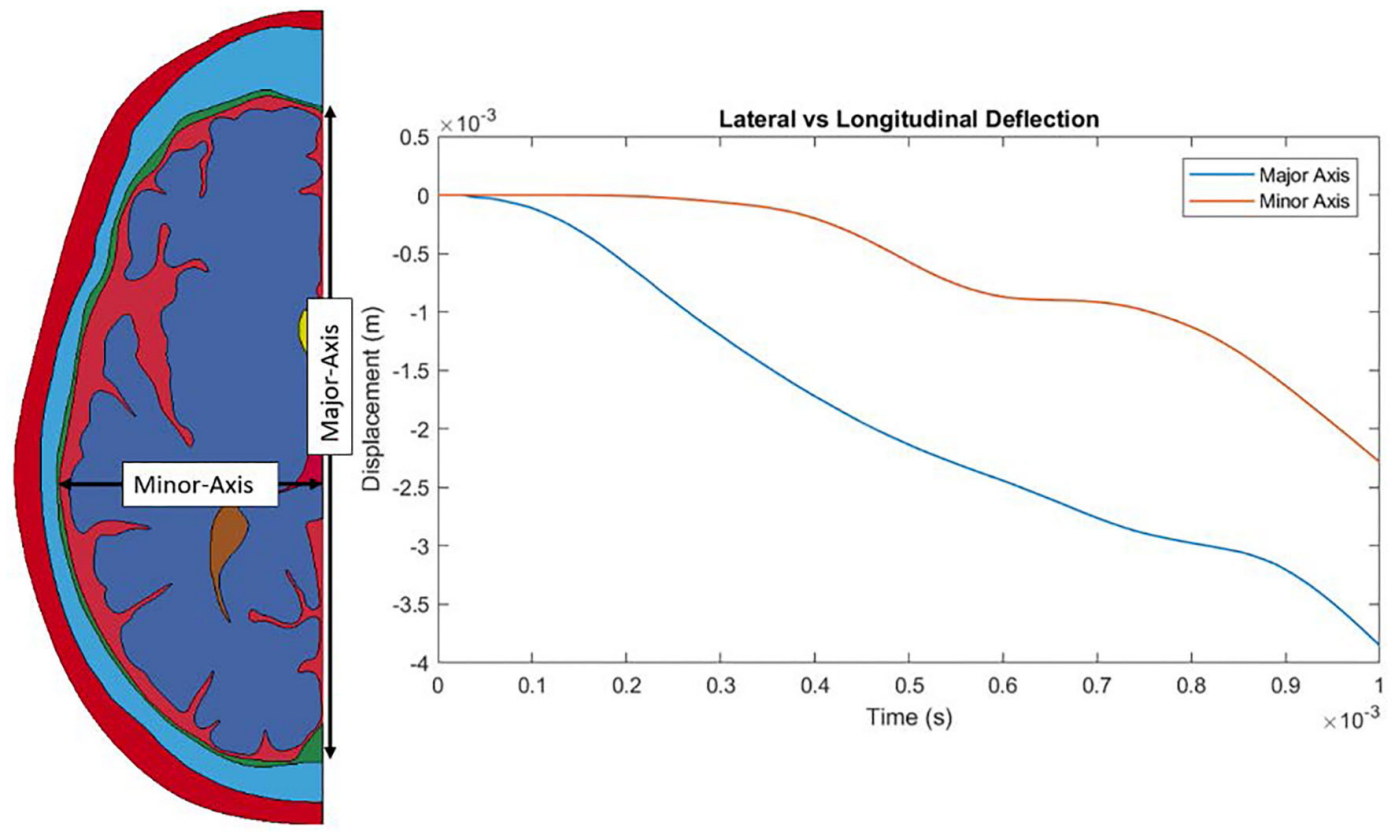
Lateral and longitudinal skull flexure throughout the simulated impact.

**Figure 6:**
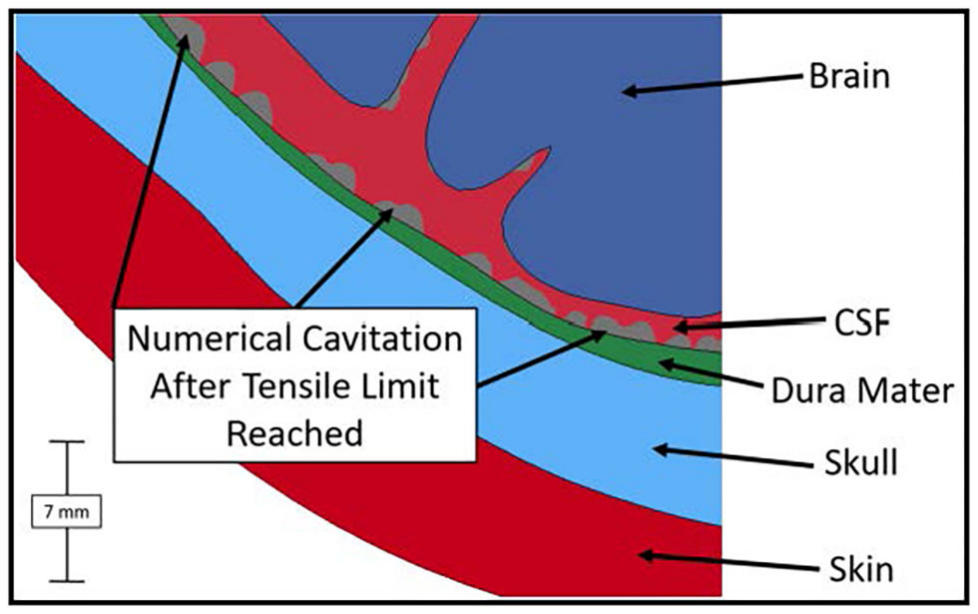
Numerical cavitation of the CSF along the posterior and lateral portions of the skull and brain when the tensile limit of CSF is reached.

Throughout the impact duration, relatively large changes to the shape of the convolutions of the brain occur. This can be seen in Figure 4, which depicts the closing of one sulcus and the partial closing of another sulcus in the anterior portion of the brain during the impact. As the sulcus closes, the CSF in the model is pushed out of the sulcus. Such closures of sulci are the reason for the inclusion of a self-contact algorithm for the brain as mentioned previously. Lateral deformation due to skull flexure are present leading to significant changes in the distance between the dura and the brain tissue as shown in Figure 5. It should be noted that the head dimension along the minor axis in the figure represents only half of the total lateral head deformation. The major axis represents total longitudinal head deformation. Additionally, the maximum longitudinal deflection is always along the major axis. The minor axis does not match the maximum lateral deflection because the position of maximum deflection changes throughout the simulation, but it does illustrate the pattern of lateral deflection. At the end of the simulation, the maximum lateral deflection is −5.12 mm while the deflection as measured by the minor axis is −2.83 mm. In response to the lateral skull flexure and motion of the brain, cavitation in the CSF is observed in both the posterior and lateral regions of the brain and interior of the skull, as shown in Figure 6. In the model all of the cavitation appear to be heterogeneous nucleation initiating at a surface between either the dura and CSF or CSF and brain with the majority originating at the Dura/CSF interface. In most engineering situation heterogeneous nucleation at a surface is more common than homogeneous nucleation within the bulk of the fluid (35). Cavitation has been experimentally observed using idealized head models at impact speeds lower than 5 m/s (36, 37).

**Figure 7:**
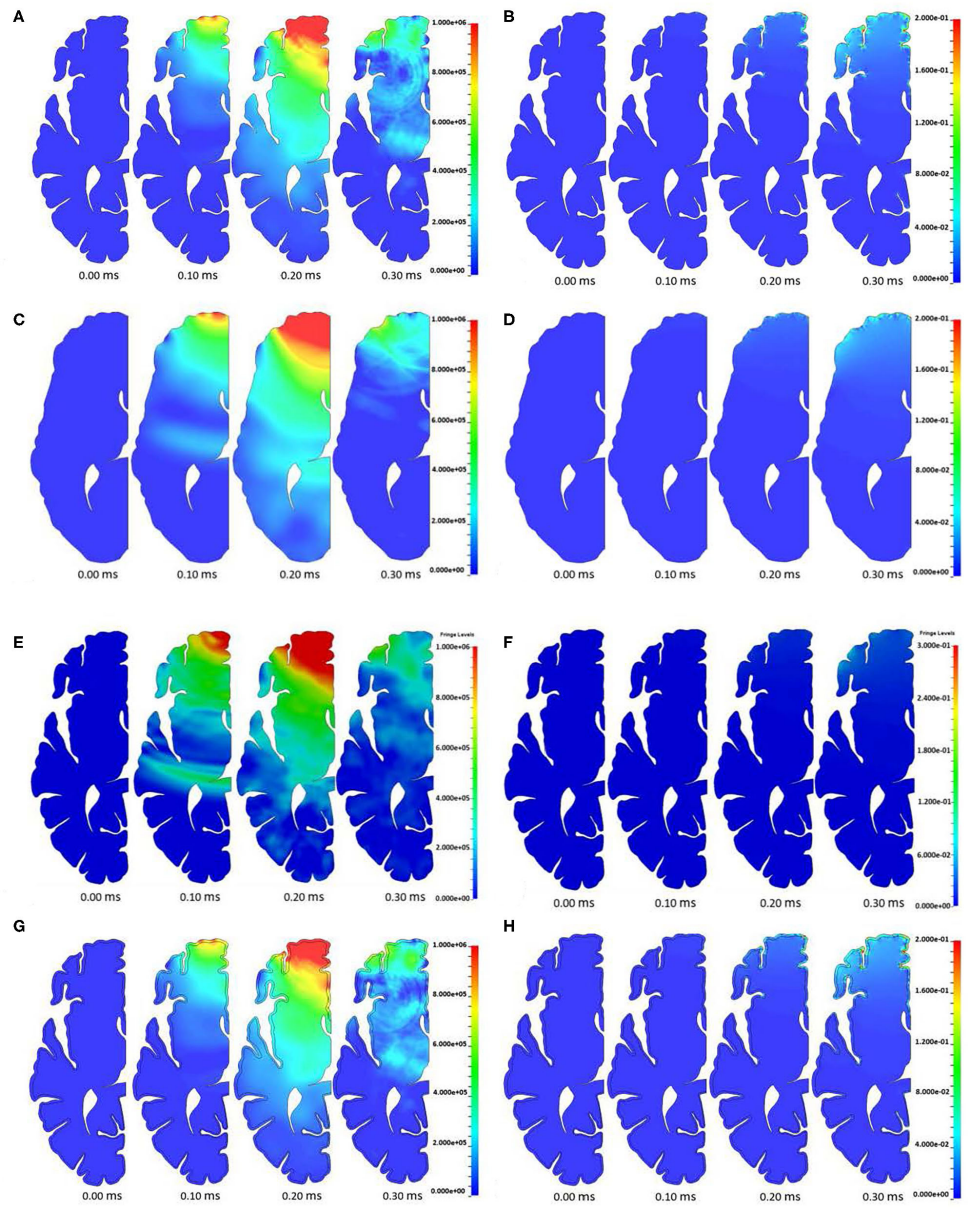
**(A)** Pressure and **(B)** shear strain evolution in the model with a homogeneous brain model. **(C)** Pressure and **(D)** shear strain evolution in the smooth brain model. **(E)** Pressure and **(F)** shear strain evolution in the Lagrangian CSF model. **(G)** Pressure and **(H)** shear strain evolution in the model with a differentiated brain model. From left to right in each grouping: 0, 0.10, 0.20, and 0.30 ms.

The time evolution of pressure in the brain is shown in Figure 7A. The pressure wave propagates from the contact region through the brain until release waves reduce the compression at a later time. No amplification of pressure at either the base of any sulcus or the top of any gyrus was observed.

**Figure 8:**
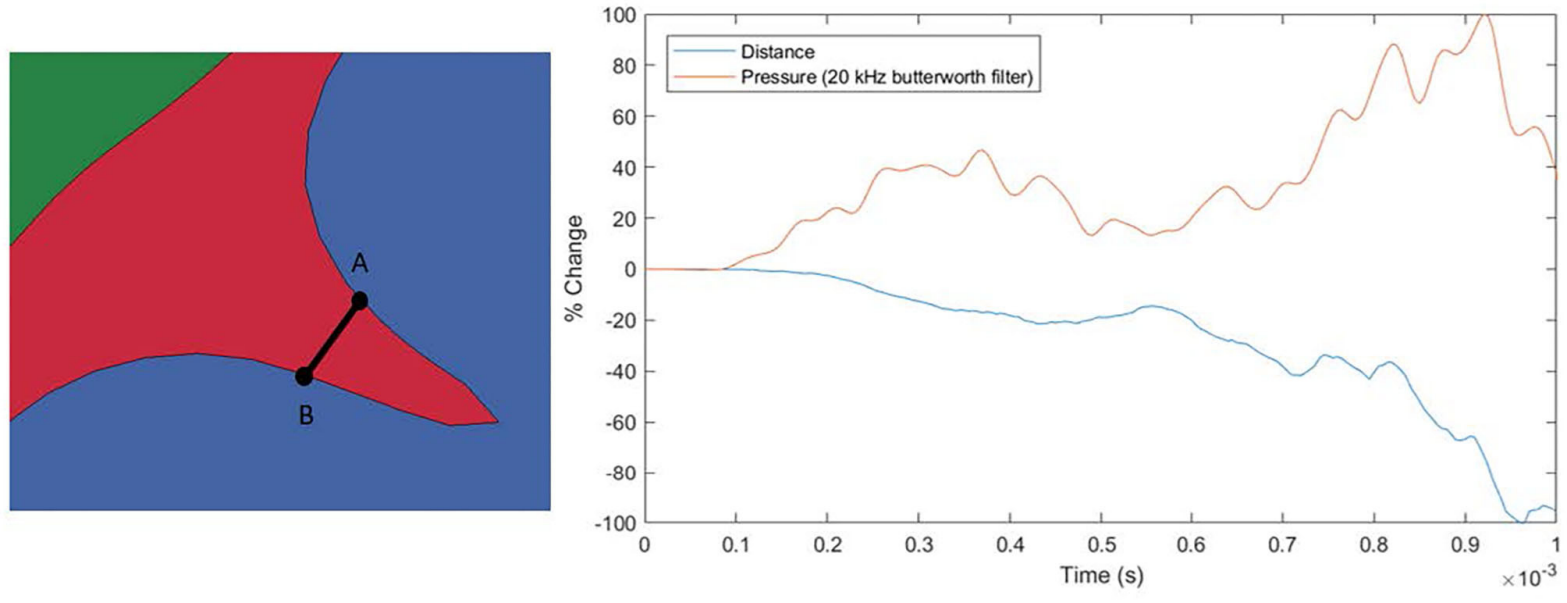
Pressure and distance plot for a sulcus. The distance is measured between points A and B with a negative value representing the points becoming closer together. Pressure is the average of the pressures at points A and B. The initial distance between A-B is ~1.3 mm.

Figure 7B shows the evolution of maximum shear strain in the brain. Specifically, Green-St. Venant maximum shear strain was analyzed in this study. It can be seen that locations with small radius of curvature exhibit elevated shear strain as the pressure wave propagates through the brain. Shear strain was examined in this study since shearing of microvessels in the brain is one of the hypothesized injury mechanisms associated with the hypothesized water hammer effect leading to TBI (1). Similarly, the first and third (both not shown) principal strains follow a similar elevated pattern at sulcal bases, as is expected due to elevation in the shear strain. Axonal stretch is another possible injury mechanism commonly associated with TBI and related to principal strains (38–40). No perceptible widening of the sulcus is apparent. An analysis of the width of the sulcus and pressure inside it indicates that the inertia of the brain, and not the pressure in the CSF, leads to the deformation of the sulcus (see Figure 8). The examined sulcus continued to close even as the pressure from the impact continued to increase. If pressure was controlling the sulcus deformation, the pressure and change in width of the sulci would be expected to be in phase with each other, which was not found to be the case. The calculated motion of the brain around this sulci appears to be consistent with the brain motion observed using high-speed x-rays and neutral density targets of impacts to cadaveric heads completed by Hardy et al. (41). At early times, Hardy et al. observed brain motion of about one mm in 5 ms for a frontal impact into a rigidly fixed block at a rate of about half of that used in this simulation (Test C383-T1). Based on the closure of the sulci shown in Figure 8, the region of the brain is undergoing a brain motion on the order of one mm in one ms. The higher rate is to be expected based on the increased impact speed. Additionally, as observed by Hardy et al. the motion in the simulated brain is lagging that of the skull. Though these two features of the simulation do not provide validation of the calculations shown in this study, it does indicate consistency between simulated and experimentally observed brain motion.

The generation of shear strain seems to arise primarily from the interaction of incident pressure wave and the included CSF–brain interface. Since our study only focuses on the time-frame when impact induced high pressure regime dominates, the possibility of a water hammer type phenomena during later relative motions between the brain and skull during the impact cannot be precluded.

We simulated the head model with the smooth brain (no gyri or sulci) approximation to assess the role of these features on stress transmission to the brain. The smooth brain approximation results are shown in Figures 7C,D. The smooth brain simulated pressure agrees reasonably well with the ALE simulation results with gyrification. However, the shear strain amplification at the base of sulci is not captured in the smooth brain approximation.

We also exercised the head model with a Lagrangian treatment of the CSF. Again, the strain amplification at the base of sulci that was captured in the ALE model of the CSF (**Figure 12**) was absent in the Lagrangian treatment of the CSF as seen in Figures 7E,F. Therefore, it is apparent that an ALE model captures higher strain amplification compared to the Lagrangian model of the CSF.

### White and Gray Matter Differentiated Model

**Figure 9:**
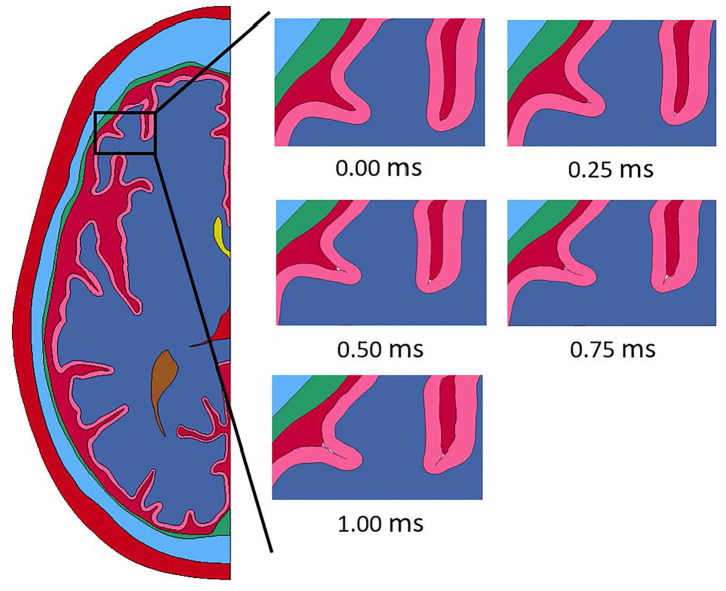
Closure of sulci throughout the duration of the impact event modeled with a differentiated brain homogeneous brain.

The deformation time history of the head model with a white and gray matter differentiated brain is shown in Figure 9. The deformation seen in the model with a differentiated brain follows all the same patterns of the model with a homogeneous brain. Namely, closure of sulci starting at the base, lateral flexure of the skull, and separation of the CSF from the dura matter along the back and side of the head.

The pressure time histories in the model with white and gray matter differentiation are shown in Figure 7G. Again, no amplification of pressure was observed at either the base of any sulcus or the top of any gyrus in this study. The addition of the layer of gray matter to the model leads to slight variations to the timing and the magnitude of the pressure wave propagation through the brain (Figures 7A,G) due to the extra interface and variation in mechanical properties between the white and gray matter.

Figure 7H shows the maximum shear strain time history of the model with differentiation between white and gray matter. Again, it can be seen that locations with small radius of curvature have elevated shear strain as the pressure waves propagate through the brain. Similarly, but not shown, the first and third principal strains follow a similar elevated pattern, as is expected due to elevation in the shear strain.

## Discussion

**Figure 10:**
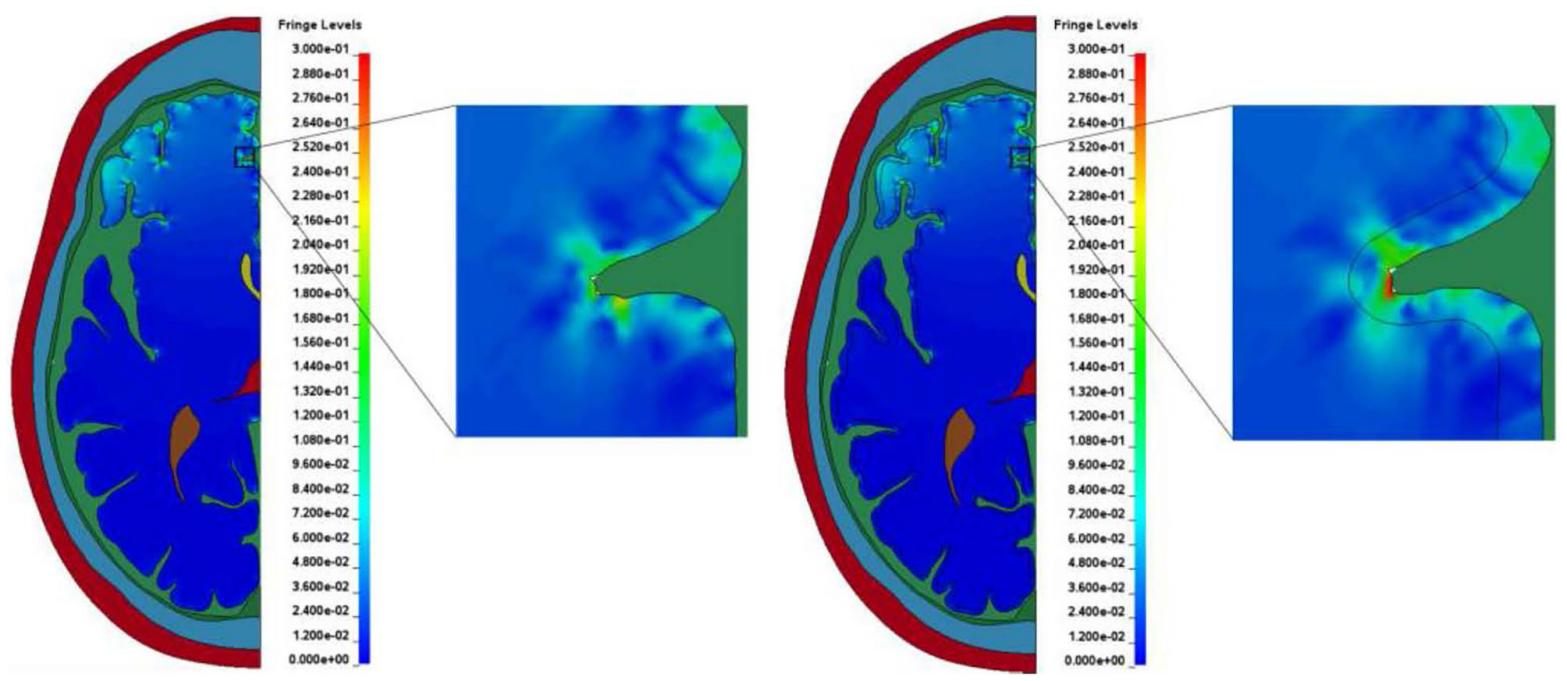
Typical strain amplification observed at the bases of sulci in the two models. **(A)** Model with homogeneous brain. **(B)** Model with white and gray matter differentiation.

Most finite element models of the human head reported in the literature include little to no gyrification of the brain and treat it as a relatively smooth part. The models presented in this study include the sulci and gyri geometry along with modeling the CSF as an ALE part in order to treat it more as a fluid. In both models presented in this study, elevated levels of shear strain and first and third principal strains near the bases of the sulci compared to the other areas in the brain are observed, as shown in Figure 10. This increased strain in the brain at the bases of sulci as they close, which would not be present in many other models, could be a potentially injurious source.

The presence of larger shear strain is noteworthy since shear stress cannot be transferred to the brain through the ALE fluid (CSF), which does not support any shear stress. We see two possible sources of the shear strain in the brain. The first mechanism is the pressure-shear conversion caused by oblique incidence of pressure waves at the CSF-brain interface. The second possibility is shear strain generation due to deformation of the brain itself, which is not impeded by the flow of CSF, especially, due to closure of the sulci. This is possible in the models presented in this study since the CSF is treated as a fluid and can be forced out of the sulci.

**Figure 11:**
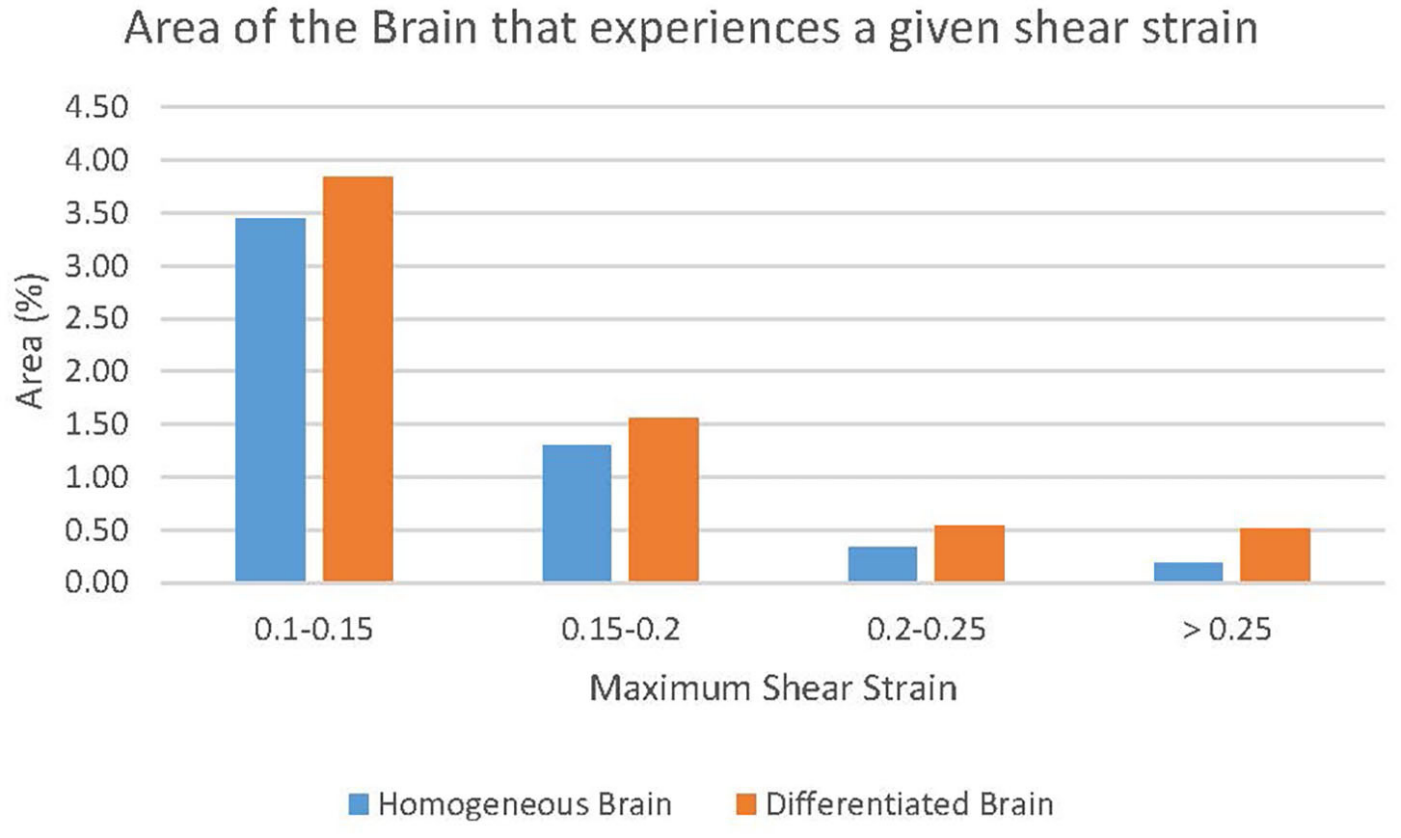
The effect of white and gray matter differentiation on maximum shear strain. The regions of the brains that exceed the highest threshold can be seen in Figure 12.

Differentiation between white and gray matter in the model led to a larger area of the brain experiencing greater maximum shear strain. This is shown by completing an analysis similar to the cumulative strain damage measure introduced by Bandak and Eppinger (38). Their measurement calculates the volume fraction of the brain, which at any time during the impact event experiences a maximum principal strain level greater than a specified threshold value. However, in this study, maximum shear strain was considered as the proposed damage mechanism under examination (1) is related to shear. Further, instead of a single threshold value for maximum shear strain several bins were used. The bins increased by 0.05 maximum shear strain until 0.25 maximum shear strain was reached. The final bin was a maximum shear strain of greater than 0.25. This analysis indicates the area of brain that experienced a maximum shear strain of greater than the lower bound of a bin but lower than the higher bound at any time during the duration of the simulated impact. The results of this measurement for both models are shown in Figure 11. In the four groupings of the greatest maximum shear strain (i.e., 0.1–0.15, 0.15–0.2, 0.2–0.25, and > 0.25) the differentiated model had a larger area of brain that experienced the strain levels. The areas of the differentiated brain that fall within the strain levels of 0.1–0.15, 0.15–0.2, 0.2–0.25, and >0.25 are 111, 120, 164, and 281% greater when compared to the areas in the homogeneous brain, respectively. This result indicates that the inclusion of a layer of gray matter in models of the human head could be necessary to capture potentially injurious mechanisms based on strain.

**Figure 12:**
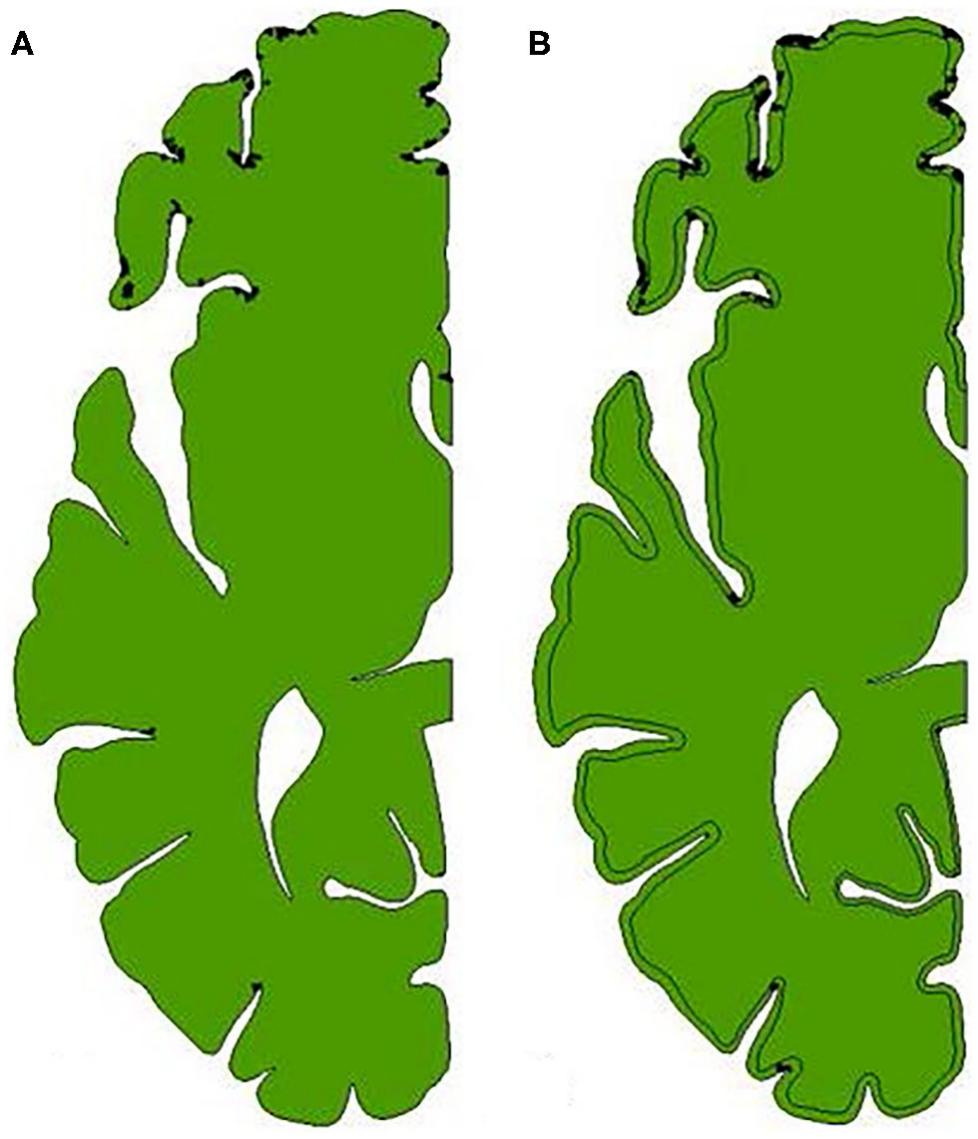
Locations in the brain that experience a maximum shear strain >0.25. Any element that is colored black has reached this threshold. The areas of the brains representing the subsequently lower bins (see Figure 11) tend to move out radially from the bases of the affected sulci. **(A)** Model with homogeneous brain. **(B)** Model with differentiated brain.

The regions of the brain that experience a maximum shear strain of greater than 0.25 during the duration of the impact for both models are shown in Figure 12. Again, the elements that experience that level of strain are largely focused near the base of sulci. The areas of the brains representing the subsequently lower bins (see Figure 11) tend to move out radially from the bases of the affected sulci.

**Figure 13:**
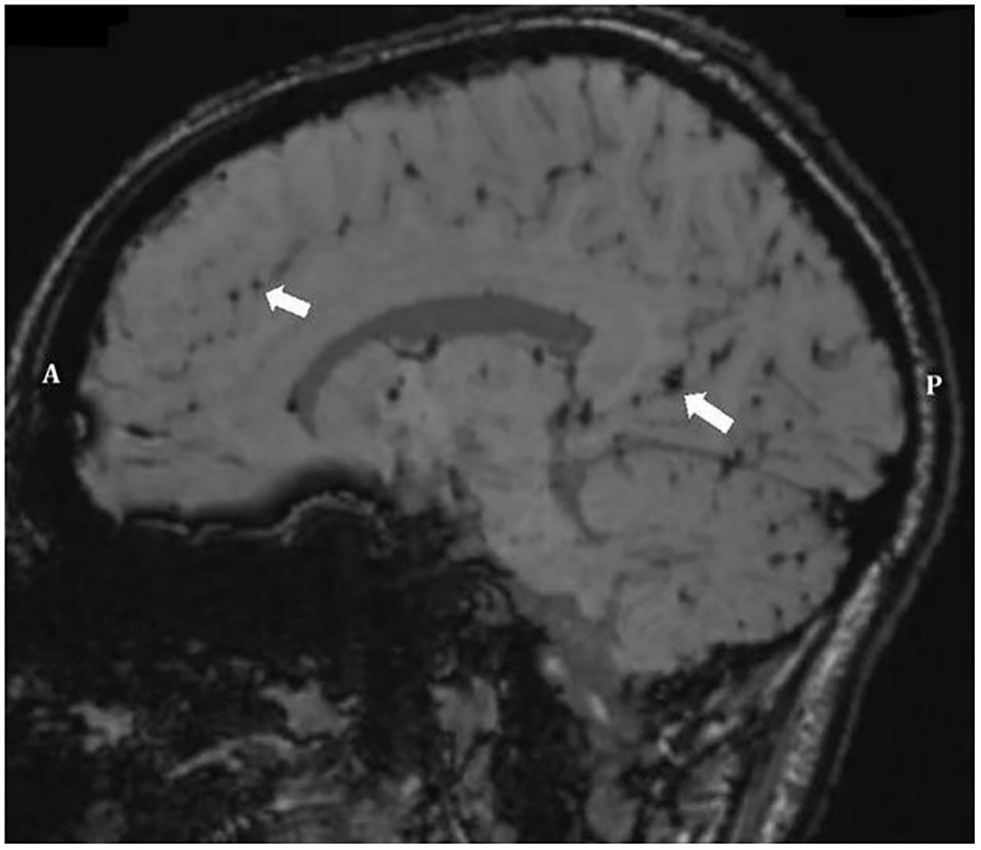
Sagittal SWI image of Player 3 (1) demonstrating multiple small areas of signal loss in the base of the sulci. Paramagnetic compounds from hemorrhages distort the magnetic field resulting in the signal loss. Arrows point to areas of micro-hemorrhage, hemosiderin, and axonal shearing/injury. Anterior (A) Posterior (P).

These regions of intense maximum shear strain near the base of the sulci compare well with the regions of micro-bleeds observed during Kornguth et al.'s study focused on female collegiate soccer players (1). Figure 13 shows a Susceptibility-Weighted Imaging (SWI) image taken as part of the study. In the figure, multiple small areas of signal loss can be seen near the base of the sulci. Paramagnetic compounds including deoxyhemoglobin, ferritin and hemosiderin from the hemorrhages distort the magnetic field resulting in the signal loss.

However, there are some limitations with the methodologies outlined in study. Several anatomical features were left out of the models in this study. These include the frontal sinuses, falx cerebri, and the variation of the skull properties through the thickness. We acknowledge the absence of these feature may influence the transition of the load to the brain. Specifially, the absence of the frontal sinuses and a skull without variation of skull properties through the thickness could attenuate the pressure wave differently. They would also alter the resultant flexure of the skull. However, the inclusion of these two features shouldn't alter the outcome of the study as they would have relatively little effect on the mechanism driving the problem (i.e., relative motion between the brain and the calvarium). The decision to not include the falx cerebri was made due to the orientation of impact being investigated and the use of 2D models. The influence of falx cerebri is most prevalent under lateral impacts and rotational events were it can influence the motion of the brain by imparting a lateral force. It would be beneficial to include this anatomical feature for the examination of other impact conditions or the development of a 3D head model that will experience rotational forces. However, the impact conditions under investigation in this study are a frontal impact with mostly translational motion of the brain, so this simplification should not change the overall trends observed in this study (23). Another limitation of the models used in this study is that there is an inherent difference in structural stiffness when using 2D model of an approximately spherical structure compared to a 3D model. This difference would be most prominent in the skull and could alter the flexure of the skull observed.

Based on the findings in this study, a broad range of mitigation strategies could be investigated to limit the observed elevated localized strains. The mitigation efforts should focus on decreasing the deceleration rate of the head and thus decreasing the motion of the brain relative to the calvarium leading to the localized strains. Mitigation technology and methodologies in several broad fields where TBI has been frequently observed should be investigated including non-contact sports, contact sports and military applications. For non-contact sports, modification of rules to minimize/limit incidental contact (e.g., prohibition of/reduction in heading of the ball in soccer) should be considered. Additionally, requiring athletes to wear soft shell padded protective gear should be investigated. In contact sports where helmets are worn with foam padding systems, optimization of the padding material response, coverage and placement for reduction in deceleration should be investigated. Active dampening technologies as an alternative to the traditional passive foam padding could be investigated. For example, the use of shear thickening fluid could provide improved helmet responses to impact loading (42). Similar helmet technologies could be considered with modification to the realm of military application.

## Conclusion

**Figure 14:**
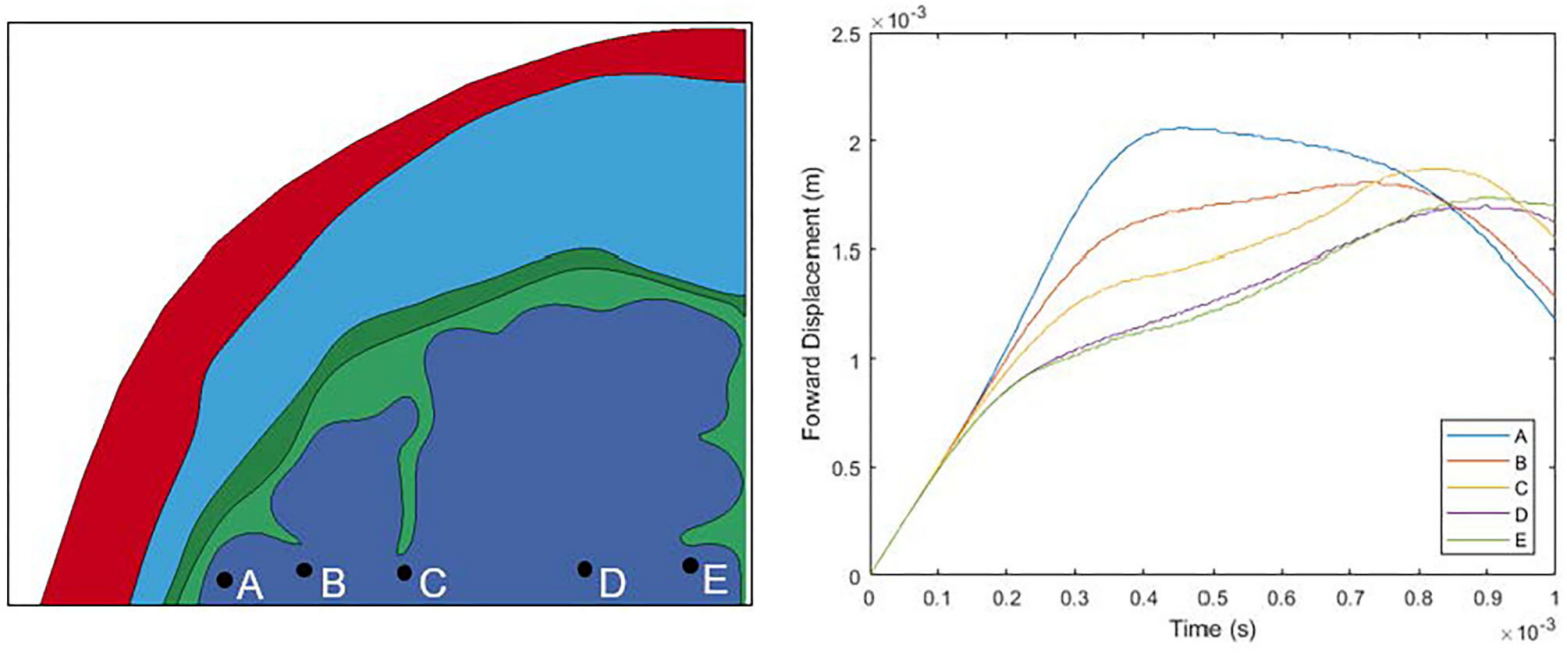
Motion of the brain throughout the duration of the simulation.

The two models presented in this study were developed to examine the proposed “water hammer hypothesis” presented by Kornguth et al. (1). The first step of the mechanism, namely CSF being driven into the sulci by the calvarium during impact, was not computationally observed in our study. It should be noted that the computational study presented in this paper focused on only the first millisecond after impact, when the impact induced high pressure dominates the response. This time frame was sufficient to capture the first relative motion between the brain and the calvarium. This can be seen in Figure 14 where the regions past the sulci examined closely in this study have stop their forward motion within the 1 ms timeframe. However, further examination of longer time frames should be conducted to determine if CSF is driven into sulci upon additional relative motions between the brain and calvarium during the impact. However, even without observing CSF driven into the sulci causing the proposed water-hammer effect, elevated maximum shear strain levels near the base of the sulci were exhibited. This correlates to the second aspect of the proposed “water hammer hypothesis,” namely shearing of tissue close to the base of the sulci especially at the interface of white and gray brain matter. The location of these regions with elevated levels of simulated strain qualitatively match the microvessel damage and microbleed patterns clinically observed in Kornguth et al.'s study (1). Further, the inclusion of differentiation between gray and white brain matter into the model increased the area of the brain that experienced high levels of maximum shear strain supporting observations of Kornguth et al. (1).

## Data Availability Statement

The datasets presented in this article are not readily available because the meshes used in the finite element simulations are based off geometry files for which each user is required by contract to have an license to access. Requests to access the datasets should be directed to the corresponding author, brian.t.fagan2.civ@mail.mil.

## Author Contributions

SK contributed a proposed the water hammer effect mechanism that would explain the localization of lesions at the base of sulci in the brain of persons subjected to rapid deceleration force associated with traumatic brain injury. The proposal resulted from his research group longitudinal study of women soccer players over a period of 7 years and also involved in interpretation of the anatomical localization of the brain region modeled in the current study. JR is the neuro-radiologist who participated in the interpretation of magnetic resonance (MR) images obtained during the study of the women soccer players and expertise in the area of traumatic brain injury assisted in the interpretation of the similarities of brain regions affected in the modeling experiments with that seen in the MR images. SS and BF developed the finite element model of the head and simulated the impacts presented in this study and partially responsible for the analysis of the simulation results. All authors contributed to manuscript revision, read and approved the submitted version.

## Conflict of Interest

The authors declare that the research was conducted in the absence of any commercial or financial relationships that could be construed as a potential conflict of interest.
